# Biochemical Reconstitution of Ca^2+^-Dependent Exosome Secretion in Permeabilized Mammalian Cells

**DOI:** 10.21769/BioProtoc.4890

**Published:** 2023-12-05

**Authors:** Jordan M. Ngo, Justin K. Williams, Isabelle M. Lehman, Randy Schekman

**Affiliations:** 1 Department of Molecular and Cell Biology, University of California, Berkeley, Berkeley, USA; 2 Department of Molecular and Cell Biology, Howard Hughes Medical Institute, University of California, Berkeley, Berkeley, USA

**Keywords:** Extracellular vesicle, Exosome, Reconstitution, Streptolysin O, Luciferase

## Abstract

Exosomes are a subpopulation of the heterogenous pool of extracellular vesicles that are secreted to the extracellular space. Exosomes have been purported to play a role in intercellular communication and have demonstrated utility as biomarkers for a variety of diseases. Despite broad interest in exosome biology, the conditions that regulate their secretion are incompletely understood. The goal of this procedure is to biochemically reconstitute exosome secretion in Streptolysin O (SLO)-permeabilized mammalian cells. This protocol describes the reconstitution of lyophilized SLO, preparation of cytosol and SLO-permeabilized cells, assembly of the biochemical reconstitution reaction, and quantification of exosome secretion using a sensitive luminescence-based assay. This biochemical reconstitution reaction can be utilized to characterize the molecular mechanisms by which different gene products regulate exosome secretion.

Key features

This protocol establishes a functional in vitro system to reconstitute exosome secretion in permeabilized mammalian cells upon addition of cytosol, ATP, GTP, and calcium (Ca^2+^).

**Graphical overview**


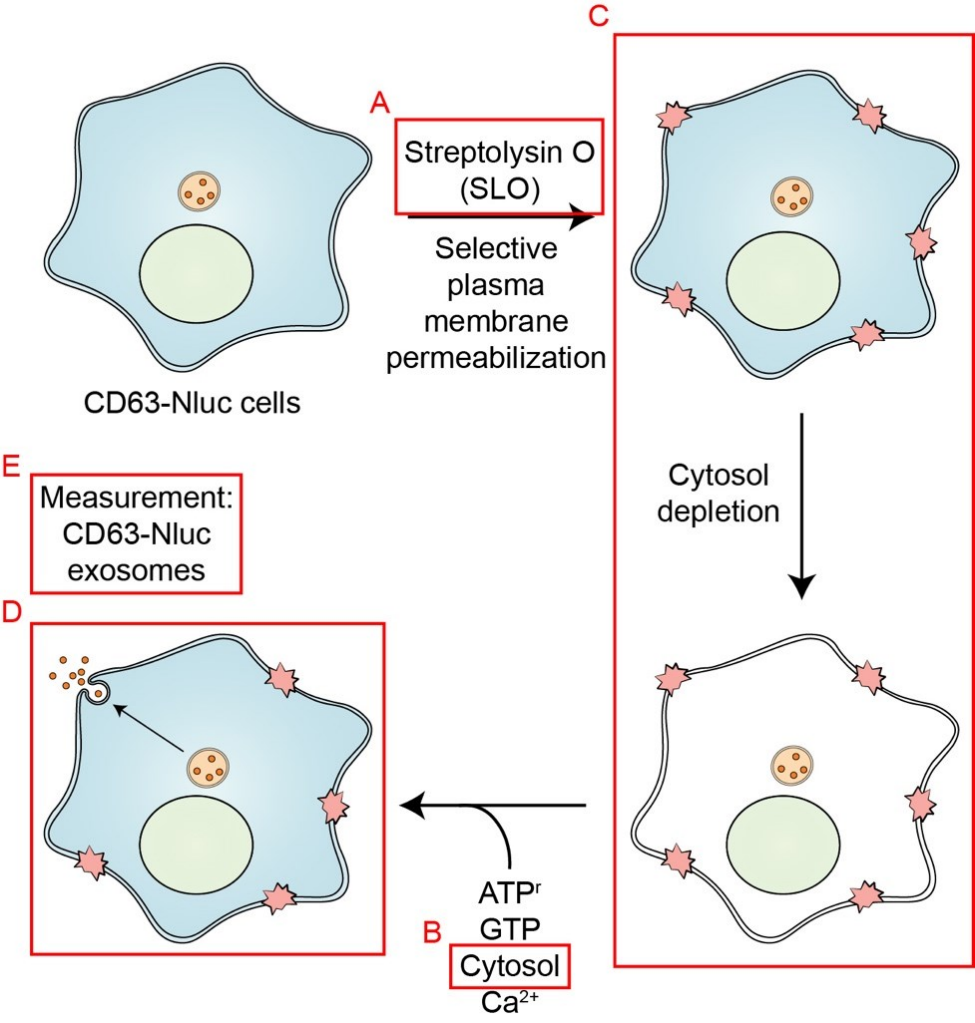


**Schematic overview of the exosome secretion biochemical reconstitution protocol.** Streptolysin O (SLO) is prepared as described in Procedure A. Cytosol is isolated from HCT116 WT cells as described in Procedure B. HCT116 CD63-Nluc cells are permeabilized by SLO as detailed in Procedure C. The assembly of the exosome secretion reactions are described in Procedure D. Quantification of CD63-Nluc secretion is detailed in Procedure E (Modified from Williams et al., 2023).

## Background

Extracellular vesicles (EVs) encompass a diverse pool of membrane-enclosed compartments released by cells to the extracellular space (Colombo et al., 2014). Exosomes are an EV subpopulation that is secreted upon fusion of multivesicular bodies (MVBs) at the cell surface and they have been suggested to play a role in intercellular communication in both physiological and disease states (Harding et al., 1983; Fong et al., 2015; Hsu et al., 2017). Of particular interest is the selective and likely tissue-specific protein and small RNA composition of exosomes, which offers the prospect of their use as biomarkers for disease progression (Shurtleff et al., 2017; Driedonks and Nolte-’t Hoen, 2019; Upton et al., 2021). Despite an accumulating interest in exosomes, the molecular mechanisms that regulate and execute their secretion are not well understood. A recent study inserted nanoluciferase (Nluc) into the endogenous locus of the exosome marker protein CD63 to allow simple quantification of exosome secretion (Hikita et al., 2018). We modified this assay by the addition of a membrane-impermeable Nluc inhibitor to allow a distinction between cellular debris and bona fide CD63-positive EVs (exosomes) (Walker et al., 2017; Williams et al., 2023). Using this assay, we demonstrated that MVBs participate in Ca^2+^-dependent plasma membrane repair. We then leveraged this modified CD63-Nluc assay to develop a biochemical reaction to reconstitute exosome secretion using cells that have been permeabilized by the bacterial pore-forming toxin, Streptolysin O (SLO). Using this biochemical reconstitution assay, we demonstrated that Annexin A6 is required for Ca^2+^-dependent exosome secretion during plasma membrane repair.

## Materials and reagents

**Biological materials**

1. HCT116 WT cells
2. HCT116 CD63-Nluc cells
*Note: Other cell lines expressing a functional and traffic-competent Nluc-CD63 fusion protein may be compatible for this reconstitution assay.*

**Reagents**

1. DMEM, high glucose, GlutaMAX^TM^ supplement (Thermo Fisher Scientific, Gibco^TM^, catalog number: 10566016)
2. Fetal bovine serum (FBS) (VWR, catalog number: 89510-194)
3. Phosphate-buffered saline (PBS), pH 7.4 (Thermo Fisher Scientific, catalog number: 10010023)
4. Liquid nitrogen
5. Protein assay dye reagent concentrate (Bio-Rad, catalog number: 5000006)
6. D-sorbitol (Sigma-Aldrich, catalog number: S1876-5KG)
7. HEPES (Sigma-Aldrich, catalog number: RDD002-1KG)
8. Potassium chloride (KCl) (Fisher Scientific, catalog number: P217-500)
9. Sodium chloride (NaCl) (Fisher Scientific, catalog number: S271-3)
10. Magnesium chloride hexahydrate [MgCl_2_·(H_2_O)_6_] (Fisher Scientific, catalog number: BP214-500)
11. Potassium phosphate monobasic (KH_2_PO_4_) (Fisher Scientific, catalog number: P285-500)
12. Potassium acetate (CH_3_COOK) (Fisher Scientific, catalog number: BP364-500)
13. Dithiothreitol (DTT) (GoldBio, catalog number: DTT25)
14. [Ethylenebis-(oxyethylenenitrilo)]-tetraacetic acid (EGTA) (Fisher Scientific, catalog number: O2783-100)
15. Calcium chloride dihydrate [CaCl_2_·(H_2_O)_2_] (EMD Millipore, catalog number: CX0130-1)
16. Adenosine-5’-triphophate (ATP) (GE Healthcare, catalog number: 27-1006-01)
17. Creatine phosphate disodium salt (Sigma-Aldrich, catalog number: 2380-25GM)
18. Creatine phosphokinase (Roche Diagnostics, catalog number: 10127566001)
19. GTP 100 mM lithium salt (Roche Diagnostics, catalog number: 11140957001)
20. Triton^®^ X-100 (TX-100) (Sigma-Aldrich, catalog number: X100-500ML)
21. Streptolysin O (SLO) (Sigma-Aldrich, catalog number: SAE0089-100KU)
22. Intracellular TE Nano-Glo^®^ substrate/inhibitor (Promega, catalog number: N2160)
  - Extracellular NanoLuc^®^ inhibitor
  - NanoBRET^TM^ Nano-Glo^®^ substrate
23. Nano-Glo^®^ Luciferase Assay System (Promega, catalog number: N1120)
  - Nano-Glo^®^ Luciferase assay substrate
  - Nano-Glo^®^ Luciferase assay buffer

**Solutions**

1. 1 M HEPES, pH 7.4 (see Recipes)
2. 1 M KCl (see Recipes)
3. 1 M NaCl (see Recipes)
4. 1 M MgCl_2_ (see Recipes)
5. 1 M KH_2_PO_4_ (see Recipes)
6. 1 M EGTA, pH 7.4 (see Recipes)
7. 1 M DTT (see Recipes)
8. 100 mM CaCl_2_ (see Recipes)
9. 10% TX-100 (see Recipes)
10. 10× ATP regeneration system (ATP^r^) (see Recipes)
11. 10 mM GTP (see Recipes)
12. 1,000× protease inhibitor cocktail (see Recipes)
13. PBS + 10 mM DTT (see Recipes)
14. PBS + protease inhibitors (see Recipes)
15. Hypotonic lysis buffer (see Recipes)
16. PBS + 1 mM EGTA (see Recipes)
17. Transport buffer (see Recipes)
18. SLO binding buffer (see Recipes)
19. Permeabilization buffer (see Recipes)
20. High-salt transport buffer (see Recipes)
21. PBS + 2% TX-100 (see Recipes)
22. Nluc substrate/inhibitor master mix (see Recipes)
23. Nluc lytic master mix (see Recipes)

**Recipes**

1. **1 M HEPES, pH 7.4 (250 mL)**
*Note: First add 200 mL of ddH_2_O, adjust pH to 7.4 with 10 N NaOH, then add ddH_2_O up to 250 mL. Store at 4 °C.*
  **Table:** 

Reagent	Final concentration	Quantity
HEPES	1 M	59.6 g
ddH_2_O	n/a	up to 250 mL
Total	n/a	250 mL
2. **1 M KCl (250 mL)**
*Note: Store at room temperature.*
  **Table:** 

Reagent	Final concentration	Quantity
KCl	1 M	18.64 g
ddH_2_O	n/a	up to 250 mL
Total	n/a	250 mL
3. **1 M NaCl (250 mL)**
*Note: Store at room temperature.*
  **Table:** 

Reagent	Final concentration	Quantity
NaCl	1 M	14.61 g
ddH_2_O	n/a	up to 250 mL
Total	n/a	250 mL
4. **1 M MgCl_2_ (250 mL)**
*Note: Store at room temperature.*
  **Table:** 

Reagent	Final concentration	Quantity
MgCl_2_·(H_2_O)_6_	1 M	50.83 g
ddH_2_O	n/a	up to 250 mL
Total	n/a	250 mL
5. **1 M KH_2_PO_4_ (250 mL)**
*Note: Store at room temperature.*
  **Table:** 

Reagent	Final concentration	Quantity
KH_2_PO_4_	1 M	34.02 g
ddH_2_O	n/a	up to 250 mL
Total	n/a	250 mL
6. **1 M EGTA, pH 7.4 (50 mL)**
*Note: First add 35 mL of ddH_2_O, adjust pH to 7.4 with solid NaOH, and then add ddH_2_O up to 50 mL.*
  **Table:** 

Reagent	Final concentration	Quantity
EGTA	1 M	19.02 g
ddH_2_O	n/a	up to 50 mL
Total	n/a	50 mL
7. **1 M DTT (10 mL)**
*Note: Make 1 mL aliquots and store at -20 °C.*
  **Table:** 

Reagent	Final concentration	Quantity
DTT	1 M	1.54 g
ddH_2_O	n/a	up to 10 mL
Total	n/a	10 mL
8. **100 mM CaCl_2_ (50 mL)**
*Note: Store at room temperature.*
  **Table:** 

Reagent	Final concentration	Quantity
CaCl_2_·(H_2_O)_2_	100 mM	0.735 g
ddH_2_O	n/a	up to 50 mL
Total	n/a	50 mL
9. **10% TX-100 (50 mL)**
*Note: Mix end-over-end to resuspend thoroughly. Store at room temperature.*
  **Table:** 

Reagent	Final concentration	Quantity
TX-100	10%	5 mL
ddH_2_O	n/a	up to 50 mL
Total	n/a	50 mL
10. **10× ATP regeneration system (ATP^r^) (20 mL)**
*Note: Make 100 μL aliquots, snap freeze in liquid nitrogen, and store at -80 °C.*
  **Table:** 

Reagent	Final concentration	Quantity
Creatine phosphate disodium salt	400 mM	2.04 g
Creatine phosphokinase	2 mg/mL	40 mg
ATP	10 mM	101.44 mg
Transport buffer	n/a	up to 20 mL
Total	n/a	20 mL
11. **10 mM GTP (4 mL)**
*Note: Make 25 μL aliquots, snap freeze in liquid nitrogen, and store at -80 °C.*
  **Table:** 

Reagent	Final concentration	Quantity
100 mM GTP	10 mM	400 μL
Transport buffer	n/a	3.6 mL
Total	n/a	4 mL
12. **1,000× protease inhibitor cocktail (10 mL)**
*Note: Make 100 μL aliquots and store at -20 °C.*
  **Table:** 

Reagent	Final concentration	Quantity
4-aminobenzamidine dihydrochloride	1 M	2.08 g
Antipain dihydrochloride	1 mg/mL	10 mg
Aprotinin	1 mg/mL	10 mg
Leupeptin	1 mg/mL	10 mg
ddH_2_O	n/a	up to 10 mL
Total	n/a	10 mL
13. **PBS + 10 mM DTT (1 mL)**
*Note: Make fresh and store at 4 °C until use.*
  **Table:** 

Reagent	Final concentration	Quantity
PBS	n/a	990 μL
1 M DTT	10 mM	10 μL
Total	n/a	1 mL
14. **PBS + protease inhibitors (25 mL)**
*Note: Make fresh and store at 4 °C until use.*
  **Table:** 

Reagent	Final concentration	Quantity
PBS	n/a	25 mL
1,000× protease inhibitor cocktail	1×	25 μL
Total	n/a	25 mL
15. **Hypotonic lysis buffer (10 mL)**
*Note: Make fresh and store at 4 °C until use.*
  **Table:** 

Reagent	Final concentration	Quantity
1 M HEPES, pH 7.4	20 mM	200 μL
1 M KCl	10 mM	100 μL
1 M EGTA, pH 7.4	1 mM	10 μL
1 M DTT	1 mM	10 μL
1,000× protease inhibitor cocktail	1×	10 μL
ddH_2_O	n/a	9.67 mL
Total	n/a	10 mL
16. **PBS + 1 mM EGTA (10 mL)**
*Note: Make fresh and store at 4 °C until use.*
  **Table:** 

Reagent	Final concentration	Quantity
PBS	n/a	10 mL
1 M EGTA, pH 7.4	1 mM	10 μL
Total	n/a	1 mL
17. **Transport buffer (50 mL)**
*Note: Make fresh and store at 4 °C until use.*
  **Table:** 

Reagent	Final concentration	Quantity
1 M HEPES, pH 7.4	20 mM	1 mL
D-sorbitol	250 mM	2.28 g
1 M KCl	120 mM	6 mL
1 M NaCl	10 mM	500 μL
1 M MgCl_2_	2 mM	100 μL
1 M KH_2_PO_4_	1.2 mM	60 μL
1 M EGTA, pH 7.4	1 mM	50 μL
1,000× protease inhibitor cocktail	1×	50 μL
ddH_2_O	n/a	up to 50 mL
Total	n/a	50 mL
18. **SLO binding buffer (1.5 mL)**
*Note: Make fresh and store at 4 °C until use.*
  **Table:** 

Reagent	Final concentration	Quantity
Transport buffer	n/a	1.5 mL
SLO	0.6 μg/mL	0.9 μg
Total	n/a	1.5 mL
19. **Permeabilization buffer (5 mL)**
*Note: Make fresh and store at 37 °C until use.*
  **Table:** 

Reagent	Final concentration	Quantity
Transport buffer	n/a	4.99 mL
1 M DTT	2 mM	10 μL
Total	n/a	5 mL
20. **High-salt transport buffer (5 mL)**
*Note: Make fresh and store at 4 °C until use.*
  **Table:** 

Reagent	Final concentration	Quantity
Transport buffer	n/a	5 mL
CH_3_COOK	1 M	0.49 g
Total	n/a	5 mL
21. **Transport buffer + 2% TX-100 (1 mL)**
*Note: Make fresh and store at 4 °C until use.*
  **Table:** 

Reagent	Final concentration	Quantity
Transport Buffer	n/a	800 μL
10% TX-100	2%	200 μL
1,000× protease inhibitor cocktail	1×	1 μL
Total	n/a	1 mL
22. **Nluc substrate/inhibitor master mix (750 μL)**
*Note: Make fresh and store at 4 °C until use.*
  **Table:** 

Reagent	Final concentration	Quantity
PBS	n/a	747 μL
Extracellular NanoLuc^®^ inhibitor	1:1,000 dilution	0.75 μL
NanoBRET^TM^ Nano-Glo^®^ substrate	1:333 dilution	2.25 μL
Total	n/a	750 μL
23. **Nluc lytic master mix (300 μL)**
*Note: Make fresh and store at 4 °C until use.*
  **Table:** 

Reagent	Final concentration	Quantity
Nano-Glo^®^ Luciferase assay buffer	n/a	294 μL
Nano-Glo^®^ Luciferase assay substrate	1:50 dilution	6 μL
Total	n/a	300 μL
**Laboratory supplies**
  1. 150 mm TC-treated cell culture dish (Corning, Falcon, catalog number: 08-772-6)
  2. 24-well BioCoat^TM^ Poly-D-Lysine (PDL) coated plates (Corning, catalog number: 356414)
  3. Amicon^®^ Ultra 15 mL centrifugal filter unit with Ultracel-3k membrane (Merck, catalog number: UFC800324)
  4. Ultra-Clear^TM^ Tube (5 mL) 13 mm × 51 mm (Beckman Coulter, catalog number: 344057)
  5. Axygen^®^ 1.5 mL Maxymum Recovery^®^ tube (Corning, catalog number: MCT-150-L-C)
  6. Posi-click 1.7 mL microcentrifuge tube [Danville Scientific, catalog number: C2170(1001002)]
  7. Cell scraper 25 cm (Sarstedt, catalog number: 83.1830) or equivalent
  8. 7 mL dounce homogenizer or equivalent
  9. AcroPrep Advance 96-well 0.4 μm filter plate (Pall Corporation, catalog number: 8029)
  10. 96-well plate, non-treated surface, non-sterile (Fisher Scientific, catalog number: 12-565-226)

## Equipment

1. Sorvall^TM^ ST16R centrifuge, TX-200 swinging bucket rotor, 400 mL round buckets, 4 × 50 mL, 9 × 15 mL conical adapters (Thermo Fisher Scientific, catalog number: 75818382)
2. Optima XE-90 ultracentrifuge (Beckman Coulter, catalog number: A94471)
3. SW 55 Ti swinging bucket rotor and bucket set (Beckman Coulter, catalog number: 342196)
4. Eppendorf 5430 R refrigerated centrifuge (Eppendorf, catalog number: 5428000015)
5. Eppendorf FA-45-30-11 fixed angle rotor (Eppendorf, catalog number: 05-401-503)
6. Eppendorf 5810 R refrigerated centrifuge with A-4-62 swinging bucket rotor and plate adaptor (Eppendorf, catalog number: 022627040)
7. Phase contrast microscope with 10× objective (any equivalent microscope works well)
8. Promega Glowmax 20/20 luminometer (any single-tube luminometer works well)

## Software and datasets

1. GraphPad Prism

## Procedure

- **Pre-activation and storage of SLO aliquots**
  1. Carefully open the bottle containing the lyophilized SLO.
  2. Add 500 μL of PBS + 10 mM DTT to the bottle.
  3. Resuspend the SLO by inverting the bottle multiple times and briefly vortexing.
  4. Pre-activate the SLO by placing the bottle within a 37 °C incubator for 2 h.
  5. Measure the protein concentration of the reconstituted SLO using the protein assay dye reagent.
  6. Snap-freeze 10 μL aliquots of pre-activated SLO in liquid nitrogen.
  7. Store at -80 °C until use in Procedure C.
*Note: Avoid repeated freeze-thaw cycles of the pre-activated SLO aliquots.*
- **Isolation of cytosol from cultured HCT116 WT cells**
  1. Culture 20 mm × 150 mm plates of HCT116 WT cells to 95% confluence in 30 mL of cell culture growth medium at 37 °C in 5% CO_2_ (DMEM, GlutaMAX^TM^ + 10% FBS).
  2. Place the cells on ice and aspirate the conditioned medium.
  3. Wash the cells with 10 mL of cold PBS per 150 mm plate.
  4. Aspirate the PBS wash buffer.
  5. Harvest the cells in cold PBS (containing protease inhibitors) using a cell scraper (1 mL of PBS per 150 mm plate) and transfer to a pre-chilled 50 mL conical tube.
*Note: This is done five plates at a time. Add 5 mL of cold PBS to one 150 mm plate, harvest the cells, and use this buffer to collect the cells with the next four plates.*
  6. Centrifuge the cells at 200× *g* for 5 min at 4 °C to sediment the cells. Discard the supernatant.
  7. Resuspend the cell pellet in 3 mL of cold hypotonic lysis buffer and incubate on ice for 15 min.
  8. Transfer the cell suspension to a pre-chilled 7 mL dounce homogenizer.
  9. Homogenize the cells using approximately 80 strokes with a tight-fitting dounce.
  10. Centrifuge at 1,000× *g* for 15 min at 4 °C in a Sorvall^TM^ ST16R centrifuge using a TX-200 swinging bucket rotor with a 15 mL conical tube adapter to sediment unruptured cells.
  11. Collect the supernatant conservatively and transfer to a 5 mL ultra-clear tube.
*Note: The volume of the post-nuclear supernatant should be ~3–4 mL. Be careful not to disturb the pellet when collecting the supernatant.*
  12. Centrifuge at ~128,000× *g* (32,500 rpm) for 30 min at 4 °C in an Optima XE-90 ultracentrifuge using a SW 55 Ti rotor to sediment cellular membranes.
  13. Collect the supernatant (cytosol fraction) conservatively and transfer to a 4 mL Amicon 3k concentrator.
  14. Centrifuge at 4,000× *g* for 6 × 10 min at 4 °C in a Sorvall^TM^ ST16R centrifuge using a TX-200 swinging bucket rotor with a 15 mL conical tube adapter to concentrate the cytosol.
*Note: Resuspend the cytosol after each 10-min centrifugation step to prevent protein precipitation. Monitor the retentate and flowthrough using the protein assay dye reagent to ensure minimal protein loss.*
  15. Collect the cytosol and measure the protein concentration using the Bio-Rad protein assay dye reagent.
*Note: The cytosol concentration should be ~40 mg/mL. This may vary depending on the cell line utilized.*
  16. Snap-freeze aliquots of cytosol in liquid nitrogen.
  17. Store at -80 °C until use in Procedure D.
*Note: Avoid repeated freeze-thaw cycles of the concentrated cytosol aliquots.*
- **Preparation of SLO-permeabilized HCT116 CD63-Nluc cells**
  1. Culture HCT116 CD63-Nluc cells to ~80% confluence in the desired number of wells within a 24-well PDL-coated plate. We typically utilize three technical replicates per experimental condition.
*Note: Do not allow cells to become overconfluent. We have observed variability in SLO permeabilization in overconfluent cultures.*
  2. Place the cells on ice and aspirate the conditioned medium.
  3. Wash each well with 500 μL of cold PBS containing 1 mM EGTA.
  4. Aspirate the PBS wash buffer and replace with 200 μL of SLO binding buffer.
  5. Gently shake at 4 °C for 15 min on a lateral shaker to allow SLO to bind to the surface of the cells.
  6. Aspirate the SLO binding buffer and wash with 500 μL of cold transport buffer to remove excess unbound SLO.
  7. Aspirate the wash buffer and add 500 μL of pre-warmed permeabilization buffer.
  8. Incubate the 24-well plate at 37 °C for 10 min to initiate cell permeabilization.
  9. Assess cell permeabilization (%) using the 10× objective of a phase contrast microscope.
*Note: The nucleoli of SLO-permeabilized cells should appear very distinct. Approximately 95%–100% of cells should be permeabilized at this stage (Figure 1).*
    **Figure 1.**
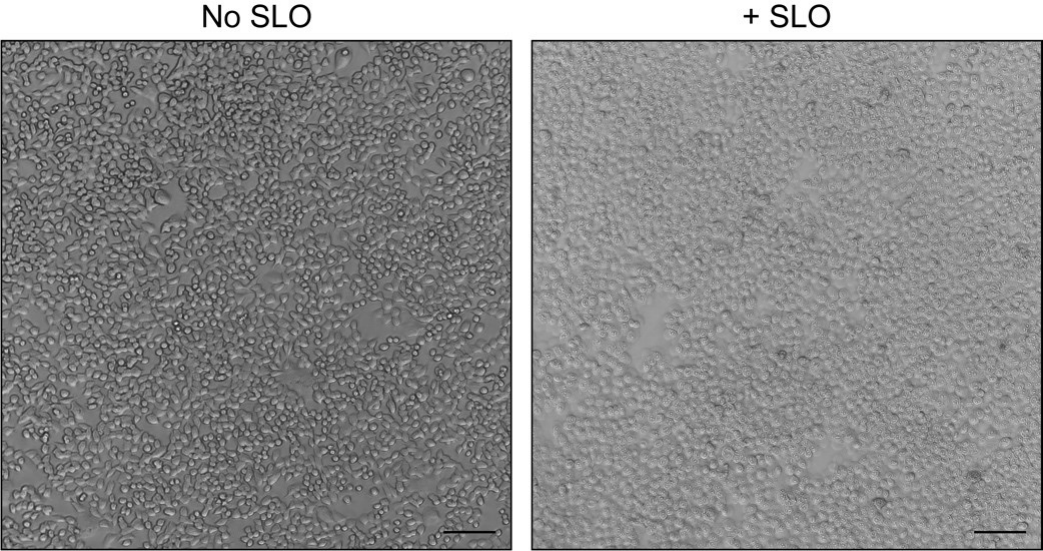
Morphology of unpermeabilized and Streptolysin O (SLO)-permeabilized cells. HCT116 CD63-Nluc cells were processed as detailed in steps C1–C9 without (left) or with (right) SLO addition. Scale bars: 100 μm.
  10. Return the 24-well plate to ice.
  11. Aspirate the permeabilization buffer, replace with 500 μL of cold transport buffer, and gently shake the 24-well plate at 4 °C for 10 min on a lateral shaker.
*Note: Start to assemble the reaction mixes for the reconstitution reactions at this time (protocol detailed in step C1 and Table 1).*
    **Table 1.** Sample calculation for biochemical exosome secretion reconstitution reactions. Each column represents a single condition for the reconstitution reaction. The total volume for each reaction mix is 250 μL for one reaction replicate and pipetting error. Each row represents the component to be added to each reaction, and the volumes indicated are in microliter. Each reaction mix can be scaled for the desired number of reaction replicates.

	(1)	(2)	(3)	(4)	(5)
**Reagent\condition**	**Membranes alone**	**Membranes + ATP^r^/GTP**	**Membranes + ATP^r^/GTP + cytosol**	**Membranes + ATP^r^/GTP + CaCl_2_**	**Membranes + ATP^r^/GTP + cytosol + CaCl_2_**
Transport buffer	250	221.2	196.2	216.2	191.2
10× ATP^r^	-	25	25	25	25
10 mM GTP	-	3.8	3.8	3.8	3.8
Cytosol (40 mg/mL)	-	-	25	-	25
100 mM CaCl_2_	-	-	-	5	5
Total	250	250	250	250	250
  12. Aspirate the transport buffer, replace with 500 μL of cold high-salt transport buffer, and gently shake the plate at 4 °C for 10 min on a lateral shaker.
  13. Aspirate the high-salt transport buffer, replace with 500 μL of cold transport buffer, and gently shake the plate at 4 °C for 10 min on a lateral shaker.
  14. Proceed immediately to Procedure D.
- **Biochemical reconstitution of Ca^2+^-dependent exosome secretion**
  1. In low retention tubes, sequentially assemble the reconstitution reaction mixes by adding the reaction components (from top to bottom). A complete 200 μL reaction contains an ATP regeneration system (1 mM ATP, 40 mM creatine phosphate, 0.2 mg/mL creatine phosphokinase), 0.15 mM GTP, 4 mg/mL cytosol, and 2 mM CaCl_2_.
  2. Aspirate the wash buffer from step C13 and replace with 200 μL of the reaction mix for each condition.
  3. Incubate the 24-well plate on ice for 5 min.
  4. Place the entire 24-well plate in a 30 °C water bath for 2 min to stimulate exosome secretion.
  5. Place the 24-well plate back on ice and immediately load 100 μL of each reaction supernatant into an AcroPrep 96-well 0.4 μm filter plate placed on top of a 96-well collection plate.
  6. Centrifuge the AcroPrep 96-well 0.4 μm filter plate at 1,500× *g* for 1 min at 4 °C in an Eppendorf 5810 R centrifuge using a A-4-62 swinging bucket rotor with a plate adaptor.
  7. While waiting on the centrifuge run, add 100 μL of cold transport buffer + 2% TX-100 (containing protease inhibitors) to each well of cells within the 24-well plate (for a final TX-100 concentration of 1%) to lyse the cells.
  8. The filtrate collected from step D6 is used to measure exosome secretion, and the lysate from step D7 is used to normalize exosome secretion between sample conditions.
- **Luminescence measurements**
  1. For the luminescence measures to quantify exosome secretion:
    - Add 50 μL of the filtrate from step D6 to a microcentrifuge tube.
    - Add 100 μL of Nluc substrate/inhibitor master mix to the filtrate.
    - Vortex the sample briefly (~1 s) and measure luminescence in a Promega Glowmax 20/20 luminometer.
*Note: This luminescence reading (Measurement A) represents CD63-Nluc luminescence obtained from intact exosomes.*
    - Remove the sample tube from the luminometer and add 1.5 μL of 10% TX-100 (for a final TX-100 concentration of 0.1%) to solubilize membranes.
*Note: This allows the membrane-impermeable Nluc inhibitor to quench any luminescence derived from membrane-protected compartments.*
    - Vortex the sample briefly (~1 s) and measure luminescence in a Promega Glowmax 20/20 luminometer.
*Note: This luminescence reading (Measurement B) represents background Nluc luminescence for each sample.*
  2. For the luminescence measurements to normalize between samples:
    - Add 50 μL of the lysate from step D7 to a microcentrifuge tube.
    - Add 50 μL of Nluc lytic master mix to the lysate.
    - Vortex the sample briefly (~1 s) and measure luminescence in a Promega Glowmax 20/20 luminometer.
*Note: This luminescence reading (Measurement C) represents total CD63-Nluc luminescence from cells and is used to normalize exosome secretion between samples.*

## Data analysis

1. The formula to calculate the exosome production index (EPI) is as follows:

EPI = [(Measurement A) – (Measurement B)]/Measurement C

**Figure 2.**
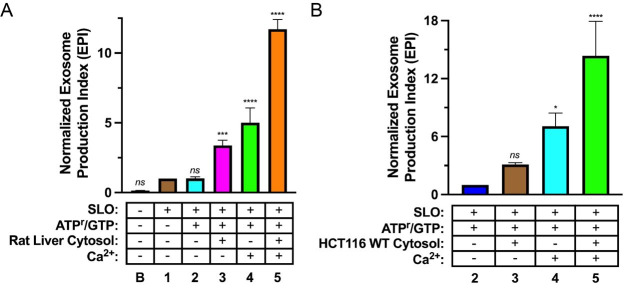
Biochemical reconstitution of Ca^2+^-dependent exosome secretion. (A–B). Exosome secretion from Streptolysin O (SLO)-permeabilized CD63-Nluc cells was assessed under different reaction conditions. The experimental conditions are indicated below each column and refer to the conditions described in Table 1 (“B” indicates baseline). Data plotted represent the means from three independent experiments, and error bars represent each standard deviation. Statistical significance was evaluated in GraphPad Prism using an ANOVA (*p < 0.05, ***p < 0.001, ****p < 0.0001, and ns = not significant) (Modified from Williams et al., 2023).

The EPI can then be normalized to the desired control condition. Example data are presented in Figure 2.

## General notes and troubleshooting

1. We have observed variability in the activity of commercial SLO preparations. We recommend titrating each batch of SLO to identify the optimal concentration required to permeabilize a majority of cells as depicted in Figure 1. If needed, trypan blue exclusion can be utilized to confirm cell permeabilization.
2. During optimization of this reconstitution assay, we observed a high level of background due to insufficient cytosol depletion. The identity and concentration of the salt utilized for the high-salt wash (step C12) may need to be optimized through empirical testing. We recommend conducting immunoblot analysis for a cytosolic marker (e.g., GAPDH) before and after the high-salt wash to ensure efficient cytosol depletion.

## References

[1] Colombo M., Raposo G. and Théry C.(2014). Biogenesis, Secretion, and Intercellular Interactions of Exosomes and Other Extracellular Vesicles. Annu. Rev. Cell Dev. Biol. 30(1): 255-289. 25288114 10.1146/annurev-cellbio-101512-122326

[2] Driedonks, T. A. P. and Nolte-’t Hoen, E. N. M.(2019). Circulating Y-RNAs in Extracellular Vesicles and Ribonucleoprotein Complexes; Implications for the Immune System. Front. Immunol. 9: e03164. 10.3389/fimmu.2018.03164 PMC6340977 30697216

[3] Fong M. Y., Zhou W., Liu L., Alontaga A. Y., Chandra M., Ashby J., Chow A., O’Connor S. T. F., Li S., Chin A. R., .(2015). Breast-cancer-secreted miR-122 reprograms glucose metabolism in premetastatic niche to promote metastasis. Nat. Cell Biol. 17(2): 183-194. 25621950 10.1038/ncb3094 PMC4380143

[4] Harding C., Heuser J. and Stahl P.(1983). Receptor-mediated endocytosis of transferrin and recycling of the transferrin receptor in rat reticulocytes. J. Cell Biol. 97(2): 329-339. 6309857 10.1083/jcb.97.2.329 PMC2112509

[5] Hikita T., Miyata M., Watanabe R. and Oneyama C.(2018). Sensitive and rapid quantification of exosomes by fusing luciferase to exosome marker proteins. Sci. Rep. 8(1): e1038/s41598-018-32535-7. 10.1038/s41598-018-32535-7 PMC6145919 30232365

[6] Hsu Y. L., Hung J. Y., Chang W. A., Lin Y. S., Pan Y. C., Tsai P. H., Wu C. Y. and Kuo P. L.(2017). Hypoxic lung cancer-secreted exosomal miR-23a increased angiogenesis and vascular permeability by targeting prolyl hydroxylase and tight junction protein ZO-1. Oncogene 36(34): 4929-4942. 28436951 10.1038/onc.2017.105

[7] Shurtleff M. J., Yao J., Qin Y., Nottingham R. M., Temoche-Diaz M. M., Schekman R. and Lambowitz A. M.(2017). Broad role for YBX1 in defining the small noncoding RNA composition of exosomes. Proc. Natl. Acad. Sci. U. S. A. 114(43): e1712108114. 10.1073/pnas.1712108114 PMC5663387 29073095

[8] Upton H. E., Ferguson L., Temoche-Diaz M. M., Liu X. M., Pimentel S. C., Ingolia N. T., Schekman R. and Collins K.(2021). Low-bias ncRNA libraries using ordered two-template relay: Serial template jumping by a modified retroelement reverse transcriptase. Proc. Natl. Acad. Sci. U.S.A. 118(42): e2107900118. 34649994 10.1073/pnas.2107900118 PMC8594491

[9] Walker J. R., Hall M. P., Zimprich C. A., Robers M. B., Duellman S. J., Machleidt T., Rodriguez J. and Zhou W.(2017). Highly Potent Cell-Permeable and Impermeable NanoLuc Luciferase Inhibitors. ACS Chem. Biol. 12(4): 1028-1037. 28195704 10.1021/acschembio.6b01129

[10] Williams J. K., Ngo J. M., Lehman I. M. and Schekman R.(2023). Annexin A6 mediates calcium-dependent exosome secretion during plasma membrane repair. eLife 12: e86556. 37204294 10.7554/eLife.86556 PMC10241516

